# The complete mitochondrial genome information of *Rana uenoi* (Amphibia, Anura, Ranidae) and the phylogenetic implication

**DOI:** 10.1080/23802359.2021.1882896

**Published:** 2021-03-01

**Authors:** Ho Young Suk, Jong Yoon Jeon, Dong-Young Kim, Sunho Cha, Mi-Sook Min

**Affiliations:** a Department of Life Sciences, Yeungnam University, Gyeongsan, South Korea; b Research Institute for Veterinary Science, College of Veterinary Medicine, Seoul National University, Seoul, South Korea; c GenoTech Corporation, Daejeon, South Korea

**Keywords:** *Rana uenoi*, Ranidae, brown frog, Korean Peninsula, phylogeny

## Abstract

We determined the complete mitochondrial genome of *Rana uenoi* (Anura: Ranidae) for the first time. The whole sequences were 17,370 bp and included 13 protein-coding genes, 2 ribosomal RNA genes, and 22 transfer RNA genes. The gene arrangement was completely identical to those observed from other Ranidae species. We used 11 protein-coding genes to examine the phylogenetic placement of this species in the genus *Rana*. *Rana* *dybowskii* was the closest sister species to *R. uenoi*. The clade of *R. uenoi* and *R. dybowskii* formed a cluster with *Rana* *huarensis*, which had a sister relationship with the group of *Rana amurensis*, *Rana coreana*, and *Rana kunyuensis*.

*Rana uenoi* is a species of brown frogs first reported in 2014. This species has previously been assigned to *Rana dybowskii* (the East Asian brown frog) that was widely distributed across Northeast China and the Far East Russia, Korean Peninsula, and Japan (Yang et al. 2017). The taxonomic controversy has long persisted regarding the complex morphological diversity of *R. dybowskii*, and a series of studies have suggested that *R. dybowskii* may be a species complex representing multiple species candidates (Matsui et al. 1998; Sumida et al. 2003; Yang et al. 2017). *Rana uenoi* was officially recorded based on evidence that populations on the Korean Peninsula and Tsushima Island showed a significant differentiation in mtDNA sequence and allozyme characteristics from individuals collected at the type locality (Vladivostok, Russia; Günther 1876) of *R. dybowskii* (Matsui 2014). This species is also clearly distinguished from the other two morphologically similar species living on the Korean Peninsula. For example, unlike *R. huanrenensis*, *R. uenoi* has paired internal vocal sacs (Matsui 2014), and the supralabial lines that *R. coreana* has distinctly are not observed in this species (Lee and Park 2016). The mitochondrial genome sequence of *R. uenoi* has not been reported to date. However, this data is truly necessary, in that questions still remain regarding the diversity and phylogenetic structure of *R. dybowskii* species complex (Yang et al. 2017).

Here, we analyzed the complete mitochondrial genome sequence of *R. uenoi*. The DNA sample was isolated from an ethanol-immersed specimen stored in the Conservation Genome Resource Bank for Korean Wildlife (CGRB: http://www.cgrb.org/) that was collected (N36°32′24.1′′/E127°50′50.01′′) in 2016. The specimen is accessible as mms7611 in CGRB. Mitogenomic sequences were extracted using the CLC genomics workbench 6.5 (http://www.qiagenbioinformatics.com/) from the reads generated from the MiSeq platform. Each mitochondrial region was annotated using the MITOS web server (Bernt et al. 2013) and manually checked using the mitochondrial information of *R. dybowskii* (NC023528.1; Li et al. 2016). The sequence information was deposited at NCBI GenBank under the accession number of MW009067. The whole sequences were 17,370 bp and included 13 protein-coding genes, 2 ribosomal RNA genes, and 22 transfer RNA genes. L-strand was observed in eight tRNA genes and ND6. Every protein-coding gene contained ATG start codon with exceptions in ND1 (GTG), COX1 (ATA), and ND4L (GTG). ND2 (TAG), COX1 (AGG), APT8 (TAA), ND4L (TAA), ND6 (AGA), and Cyt *b* (TAA) were terminated with the stop codons of vertebrate mitochondrial DNA. An incomplete stop codon was detected at the remaining six coding genes. The gene arrangement was completely identical to those observed from *R. dybowskii* (Li et al. 2016). We used 11 protein-coding genes (8964 bp; excluding ND5 and ND6 to avoid incomplete alignment) to examine the phylogenetic placement of this species in the genus *Rana* (Figure 1). *Rana dybowskii* was the phylogenetically closest sister species to *R. uenoi*. The clade of *R. uenoi* and *R. dybowskii* formed a cluster with *Rana huarensis*, which had a sister relationship with the group of *Rana amurensis*, *Rana coreana*, and *Rana kunyuensis* (Figure 1). Our study can provide useful information to reconstruct the consensus phylogenetic tree that is essential for the future study of brown frog diversity.

**Figure 1.**
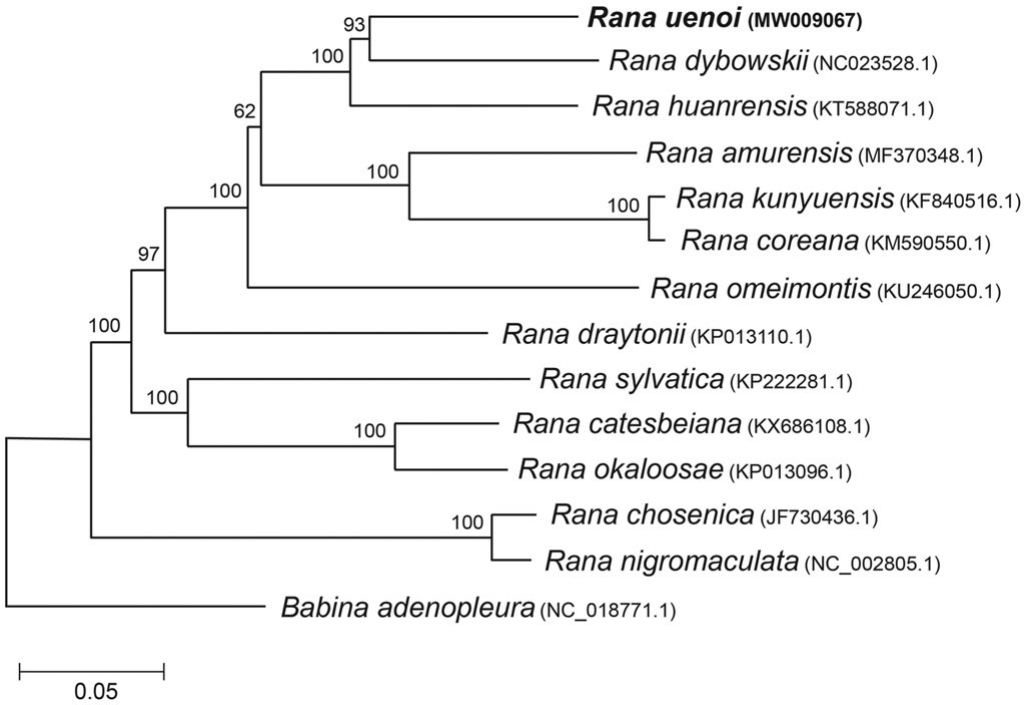
Phylogenetic tree generated using the neighbor-joining method (1000 bootstrapping, MEGA 7) based on eleven mitochondrial coding genes of 13 *Rana* species.

## Data Availability

The genome sequence data that support the findings of this study are openly available in GenBank of NCBI at (https://www.ncbi.nlm.nih.gov/) under the accession no. MW009067. The associated BioProject, SRA, and Bio-Sample numbers are PRJNA689962, SRR13364873, and SAMN17222395, respectively.
